# Use of Shockwave Lithoplasty for a Patient With Recurrent Angina

**DOI:** 10.7759/cureus.14823

**Published:** 2021-05-03

**Authors:** Sabah Siddiqui, Sergey Ayzenberg, Ahmad Morshed, Mazin Khalid, Samantha Ehrlich

**Affiliations:** 1 Cardiology, Maimonides Medical Center, Brooklyn, USA; 2 Cardiology, Maimonides Medical Center, New York, USA; 3 Internal Medicine, Maimonides Medical Center, Brooklyn, USA

**Keywords:** coronary calcium lesions, in-stent restenosis, ifr, angina, lithoplasty

## Abstract

Current calcium modification treatments only address the burden of intimal calcium with varying degrees of success and result in an increased risk for adverse events. Here, we describe the use of shockwave intravascular lithoplasty (S-IVL) to effectively treat a severely calcified coronary artery lesion. A 59-year-old male with a history of coronary artery disease with stents presented to our hospital with angina. Diagnostic coronary angiography revealed a mid-right coronary artery (mRCA) stent with severe in-stent restenosis due to under expansion of stent with severe calcification. Due to these factors, the decision was made to reduce the calcium burden with the use of S-IVL. This is a promising technique in plaque modification of severely calcified coronary lesions with less risk of myocardial injury and mechanical vascular trauma. It is important to customize the choice of therapy based on the patient and the characteristics of the coronary lesion.

## Introduction

Moderate to severe calcification in coronary culprit vessels is a strong predictor of future major adverse cardiovascular events (MACE) [1]. Coronary artery stenosis with calcifications is difficult to treat as the calcium deposits make the use of drug-coated balloons and stents less effective [2,3]. Coronary calcification can impair device delivery, damage the drug-eluting polymer of the stent, impair stent apposition and inhibit expansion, thus predisposing to stent failure, and result in stent thrombosis and re-stenosis [4-7] Current calcium modification treatments, which can be difficult to perform, address the burden of intimal calcium with varying degrees of success and result in an increased risk for adverse events [8]. Heavily calcified lesions must be optimally prepared before percutaneous coronary intervention (PCI) and it is important to customize the choice of therapy based on the specifics of the coronary lesion. Here we describe a unique case where we successfully used S-IVL to treat a patient with unstable angina with a severely calcified coronary artery lesion with multiple layers of stents.

## Case presentation

**Figure 1:**
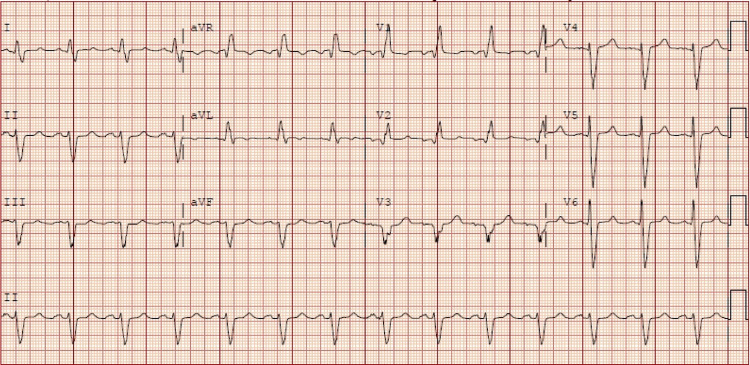
Electrocardiogram showing normal sinus rhythm with left atrial enlargement, right bundle branch block, and left anterior fascicular block

A 59-year-old male with a past medical history of ischemic cardiomyopathy with stents, and a current smoker presented to our hospital with complaints of exertional, substernal, crushing chest pain. On presentation, his electrocardiogram showed normal sinus rhythm with left atrial enlargement, right bundle branch block and left anterior fascicular block (Figure 1) and peak troponin of 0.22 (normal 0.00-0.02 ng/mL).

**Figure 2:**
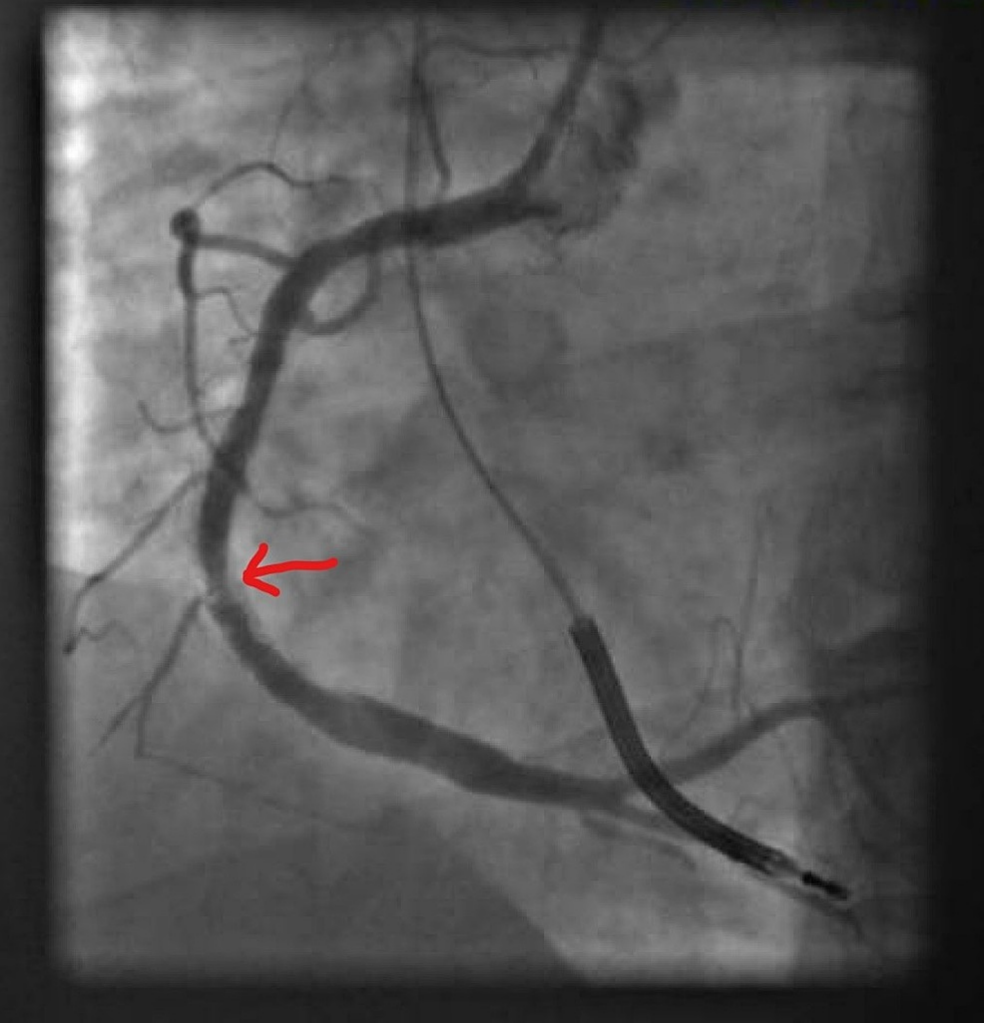
Diagnostic CAG with red arrow showing severe 95% in-stent restenosis of mRCA stent CAG: Coronary angiography mRCA: mid-right coronary artery

**Figure 3:**
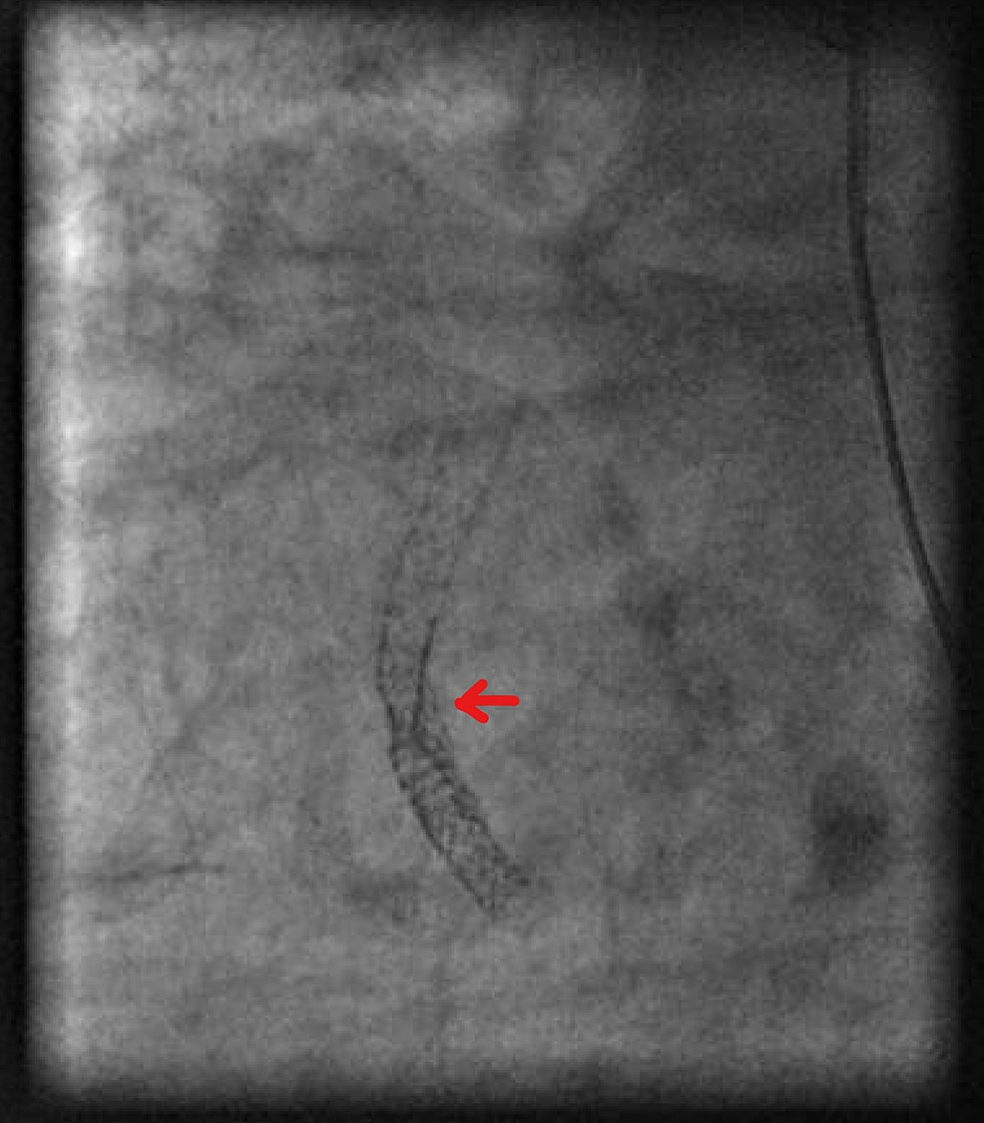
CAG showing in-stent restenosis of mRCA due to under expansion of stent (red arrow) CAG: coronary angiogram mRCA: mid-right coronary artery

Coronary angiogram (CAG) revealed severe in-stent restenosis in the mid-right coronary artery (mRCA) (Figure 2) due to under-expansion of the stent (Figure 3).

**Figure 4:**
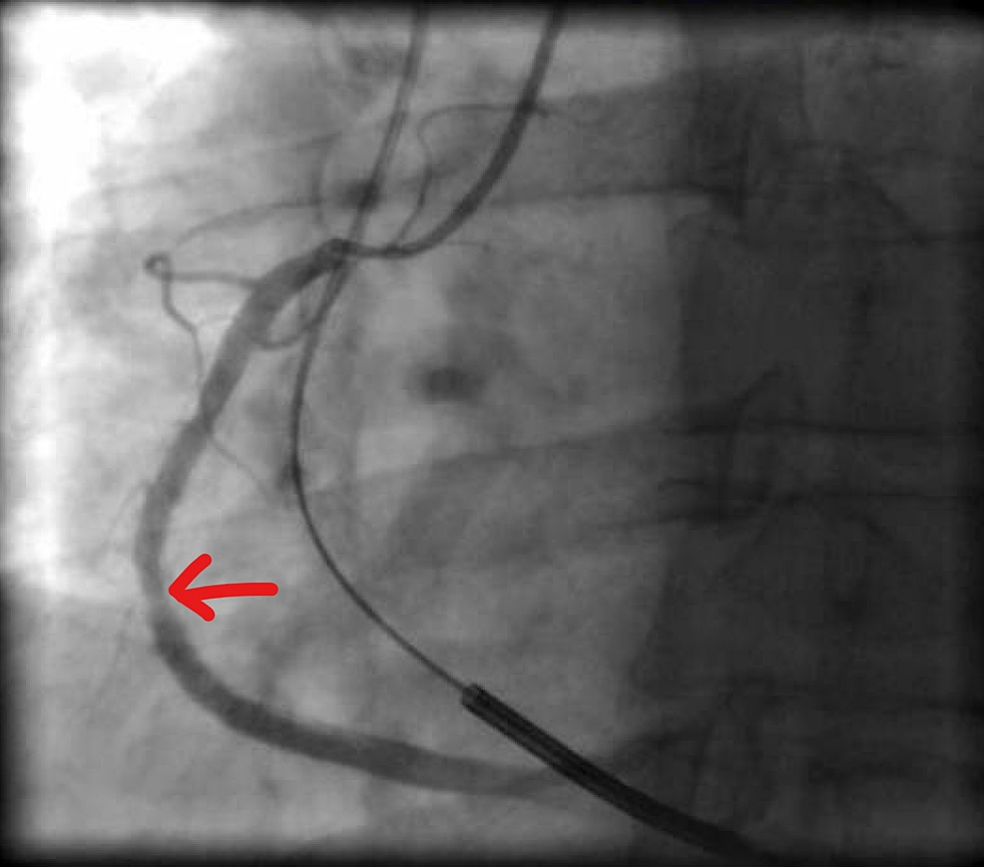
CAG from January 2018 showing mRCA after being treated with an Angiosculpt scoring balloon CAG: coronary angiogram mRCA: mid-right coronary artery

The mRCA had a previous Cypher 3.5 mm x 33 mm (Cordis, CA) drug-eluting stent (DES) placed in 2004. There was evidence of mild in-stent restenosis in 2009 when the patient received a mid-left anterior descending (LAD) stent at that time. A second Synergy Rx 4.0mm x 16 mm DES (Boston Scientific, MA) was placed in 2017. In January 2018, in-stent restenosis of mRCA was treated using a 3.5 mm x 15 mm Angiosculpt (Spectranetics, CO) scoring balloon (Figure 4).

**Figure 5:**
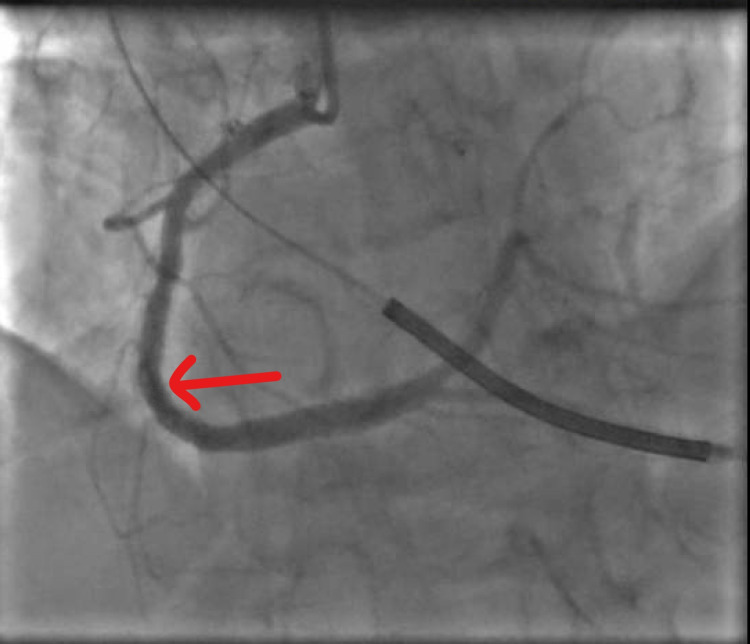
CAG showing mRCA post PTCA and shockwave lithotripsy CAG:  Coronary angiography mRCA: mid-right coronary artery PTCA: percutaneous transluminal coronary angioplasty

Fractional flow reserve (FFR) is an invasive physiologic index that uses hyperemic stimulation and maximal vasodilation to assess the functional significance of coronary stenosis. Instantaneous wave-free ratio (iFR) is another physiological index that does the same, but can be obtained at rest without hyperemic stimulation. Both are an accepted reference standard to indicate whether a stenosis is likely to be responsible for ischemia. Stenosis with an ischemic value of FFR and iFR should be revascularized as they are associated with symptoms and worse outcomes, but lesions with a non-ischemic FFR and iFR can be treated medically and have a more favorable prognosis [9]. Once we had obtained diagnostic images, an iFR was obtained at 0.66 (iFR < 0.89 suggestive of flow restriction) and FFR was 0.76 (FFR > 0.80 excludes inducible ischemia). The lesion itself was focal, concentric, and heavily calcified. The lesion was predilated; however, in the presence of under-expanded, multiple layers of stents, and significant calcification in the lesion, the decision was made to reduce the calcium burden by using Shockwave Lithoplasty (Shockwave Medical, CA) [10]. A 7-French JR4 guiding catheter (Medtronic, MN) was used to engage the RCA and an Extra-Sport 0.014 coronary guidewire (Abbott, IL) was used to cross the lesion. We used a 5-French Shockwave S4 device (Shockwave Medical, CA) with a 4.0 mm x 40 mm IVL balloon-catheter platform to perform lithoplasty to target and dilate the lesion. Twenty pulses of 10 sec of ultrasound energy were applied through the balloon which was first inflated to 2 atm and then 4 atm. It was not inflated any further to avoid the risk of potential shockwave balloon perforations. The result was optimized with a 4.0mm x 8mm non-compliant Emerge over-the-wire balloon (Boston Scientific, MA) at 16 atm, which resulted in a TIMI 3 flow (Figure 5).

Post-intervention FFR was performed with a value of 0.90 after adenosine was administered. The patient tolerated the procedure well, was asymptomatic and subsequently discharged home. The patient remains chest pain free after more than 10 months of outpatient follow up.

## Discussion

Lithoplasty (Shockwave Medical, CA) is a technology based on lithotripsy that combines a balloon angioplasty catheter with the use of sound waves, similar to that used for kidney stones [2]. The Shockwave Medical Lithoplasty System (Shockwave Medical, CA) was originally designed to be delivered through the peripheral arterial system of the lower extremities to the site of a calcified lesion. With the recent advances in its use for coronary calcific lesions, we wish to elaborate on our own successful experience to establish a clinical discussion. The S-IVL device is a single-use, monorail catheter with a central ultrasound core delivered over a 0.014˝ angioplasty wire which contains lithotripsy emitters in an integrated balloon that deliver pulses of sound waves throughout the interior of the artery wall to break up superficial and deeper calcifications before the angioplasty balloon inflation and improve plaque compliance [5,8,11]. Each lithotripsy cycle is 10 s at a frequency of one pulse per second. Each catheter can perform eight cycles of lithotripsy [11].

Current techniques to treat calcific stenosis include standard or high-pressure non-compliant, cutting, scoring balloons, or rotational atherectomy. There is a potential for increased adverse events such as vascular wall injury and coronary dissection or perforation with these techniques [6]. Rotational or orbital atherectomy can favorably modify coronary artery calcification (CAC) to allow for delivery of interventional devices, but the plaque modification itself may be limited by guidewire bias [12]. Atherectomy, including laser, can also induce arterial injury by the generation of heat and cause myocardial injury with or without the clinical manifestation of slow flow or no flow as it microembolizes the atherosclerotic material [13]. Specialty balloons using cutting or scoring technologies may avoid these complications, but, the available literature demonstrated the lack of benefits in restenosis or MACE and an increased risk of myocardial infarction (MI) and vessel perforation with specialty balloons [12].

Intravascular lithrotripsy is balloon based, and as a result, the risk of atheromatous embolization is lower than with the use of free debulking devices. Plaque modification using IVL is not subject to guidewire bias; additionally, side-branch protection using a guidewire may be easily performed using IVL, without risk of wire entrapment or severing as may occur with rotational or orbital atherectomy. Energy is distributed uniformly across the lithotripsy emitter addressing calcium irrespective of its circumferential location [12]. The Lithoplasty system is intended to fracture calcifications with the use of lower pressure balloon expansion and delivers pulsatile sonic pressure waves locally to break down the intimal and medial calcium in the artery wall with minimum trauma to the artery [14]. This also allows for increased vessel compliance and optimizes stent expansion [11]. It offers safe calcium debulking even through two layers of stent struts [10].

The Disrupt PAD III Observational Study had demonstrated the use of peripheral IVL to treat severely calcified, stenotic peripheral artery disease (PAD) with low residual stenosis, high acute gain, and a low rate of complications [13]. The Disrupt CAD I study was the first to demonstrate the feasibility of IVL as a new therapeutic modality for management of severe CAC. Following this, the Disrupt CAD II study confirmed that in patients with severe CAC who require coronary revascularization, IVL was safely performed with high procedural success, minimal complications and resulted in substantial calcific plaque fracture in most lesions [12]. Disrupt CAD III also confirms the safety and effectiveness of coronary IVL facilitated stent implantation in severely calcified lesions. There were no perforations seen in the trial and although imaging is not required, optical coherence tomography and calcium volume index may be useful when confronted with heavily calcified vessels. In addition, the use of scoring/cutting balloons or atherectomy, while not used in the trial may be helpful in cases of severe calcific lesions [15]. Finally, a small case series by Wong B et al. established that when PCI for STEMI is complicated by the presence of heavy calcification, the S-IVL technique appears to be a safe and straightforward option to help achieve procedural success [11].

It is pertinent to be aware of the potential complications of S-IVL. There has been an incidence of IVL balloon bursting during lithrotripsy therapy resulting in a vessel dissection. There may be reduced efficacy of therapy due to inadequate balloon apposition to the vessel wall in vessels of more than 4mm (maximum shockwave balloon size) [8]. The IVL system may be inappropriate or need to be used cautiously if critical stenosis or severe vessel tortuosity is present.

## Conclusions

Severe coronary calcification can create difficulties for stent implantation and predispose to stent failure, in-stent thrombosis and restenosis. While physiological tools FFR and iFR were used in our above case, intraluminal coronary imaging such as intravascular ultrasound (IVUS) or optical coherence tomography (OCT) may also be helpful diagnostic and management tools. Although there is demonstratable success in the utility of lithoplasty in the management of a heavily calcified hemodynamically significant stenosis such as in the case of our patient, further studies may be needed to examine the benefits of IVL in such complex PCI and unique situations of coronary calcification. This article highlights the usefulness in certain scenarios such as in-stent restenosis with severely calcific lesions, but more importantly, creates the base for clinical discussion.
